# Transcriptome Response of Liver and Muscle in Heat-Stressed Laying Hens

**DOI:** 10.3390/genes12020255

**Published:** 2021-02-10

**Authors:** Yan Wang, Xinzheng Jia, John C. F. Hsieh, Melissa S. Monson, Jibin Zhang, Dingming Shu, Qinghua Nie, Michael E. Persia, Max F. Rothschild, Susan J. Lamont

**Affiliations:** 1 Department of Animal Science, Iowa State University, Ames, IA 50011, USA; wynew2004@163.com (Y.W.); xinzhengjia@fosu.edu.cn (X.J.); jhsieh@iastate.edu (J.C.F.H.); msmonson@iastate.edu (M.S.M.); jibzhang@coh.org (J.Z.); mfrothsc@iastate.edu (M.F.R.); 2 State Key Laboratory of Livestock and Poultry Breeding, Guangdong Key Laboratory of Animal Breeding and Nutrition, Institute of Animal Science, Guangdong Academy of Agricultural Sciences, Guangzhou 510640, China; shudm@263.net; 3 School of Life Science and Engineering, Foshan University, Foshan 528225, China; 4 Toni Stephenson Lymphoma Center, City of Hope, Duarte, CA 91010, USA; 5 College of Animal Science, South China Agricultural University, Guangzhou 510642, China; nqinghua@scau.edu.cn; 6 Department of Animal and Poultry Sciences, Virginia Tech, Blacksburg, VA 24061, USA; mpersia@vt.edu

**Keywords:** RNA-Seq, pectoralis major muscle, liver, layer chickens, heat stress

## Abstract

Exposure to high ambient temperature has detrimental effects on poultry welfare and production. Although changes in gene expression due to heat exposure have been well described for broiler chickens, knowledge of the effects of heat on laying hens is still relatively limited. In this study, we profiled the transcriptome for pectoralis major muscle (*n* = 24) and liver (*n* = 24), during a 4-week cyclic heating experiment performed on layers in the early phase of egg production. Both heat-control and time-based contrasts were analyzed to determine differentially expressed genes (DEGs). Heat exposure induced different changes in gene expression for the two tissues, and we also observed changes in gene expression over time in the control animals suggesting that metabolic changes occurred during the transition from onset of lay to peak egg production. A total of 73 DEGs in liver were shared between the 3 h heat-control contrast, and the 4-week versus 3 h time contrast in the control group, suggesting a core set of genes that is responsible for maintenance of metabolic homeostasis regardless of the physiologic stressor (heat or commencing egg production). The identified DEGs improve our understanding of the layer’s response to stressors and may serve as targets for genetic selection in the future to improve resilience.

## 1. Introduction

Heat stress has negative impacts on agricultural animal production as it reduces the animal’s ability to dissipate body heat and thus deteriorates efficiency of energy utilization by animals [1]. Due to their rapid body growth and greater metabolic activity, commercial poultry have become particularly sensitive to temperature-associated environmental challenges [2,3,4]. The detrimental effects of heat stress on broilers and laying hens ranges from reduced growth and egg production to decreased meat and egg quality and food safety [4]. In layers, heat stress negatively affects feed intake, endocrine function, acid-base balance, and organ functions; this can result in lower egg production and decreased welfare for laying hens [5,6,7,8]. Genetic influence on response to heat stress in layer chickens has been shown by different survivability of two layer populations divergently selected for heat tolerance [9]. Therefore, understanding heat stress responses and improving heat tolerance at the genetic level is a feasible permanent approach towards lessening the negative effects of high environmental temperatures on chicken performance and welfare.

The impact of heat stress depends not only on the tolerance of chickens but also on duration of exposure. For example, in plasma from broiler chickens, acute heat stress can decrease triglyceride, creatine kinase (CK) and triiodothyronine levels, while chronic heat stress can increase lactate dehydrogenase, glutamic-oxaloacetic transaminase and CK, which are indicators of tissue damage [10]. In addition, transcriptome analysis by RNA sequencing (RNA-Seq) also revealed differential responses to acute and chronic heat stress through large differences in the number of differentially expressed genes (DEGs) in broiler liver transcriptome [11]. Among chicken heart, liver and pectoralis major muscle, muscle was the least protected during heat stress, as indicated by increased protein oxidation without changes in expression of heat shock transcription factors (HAFs) and heat shock proteins (HSPs) [12].

Previous studies of muscle tissue under heat stress have been mostly conducted in broilers, as adverse effects of heat stress are more evident in large-bodied, rapidly growing meat-type chickens than in layer-type chickens [13,14]. In the transcriptome profile for the muscle tissue for both types of chickens, more gene expression changes of heat-stressed compared to non-heat-stressed birds were observed for the broilers [15]. Similarly, the liver has been found to be susceptible to oxidative stress in broilers under heat stress [16,17]. Changes in transcriptome expression profile of the liver under heat stress has been reported in broilers [11,18,19,20,21]. In laying hens, effects of heat stress have been identified on egg production parameters, immune responses, ovarian function, and metabolic activity [19,22,23]. Modulation of the metabolism in the muscle and liver to maintain organismal homeostasis under stress may have an impact on egg production.

To our knowledge, there have been no RNA-Seq experiments characterizing whole transcriptome changes in muscle or liver for sexually mature laying hens under acute and chronic heat stress. In the present study, we utilized RNA-Seq to characterize gene expression changes for both pectoralis major muscle and liver from layers early in their egg-production cycle at three time points during a 4-week cyclic heat challenge. Characterizing gene expression responses to heat stress during this crucial period will help us to understand the impact of heat stress on physiological changes. Through comparisons of the transcriptomes from heat-treated and control layers, we aim to identify important genes and potential mechanisms involved in metabolic regulation as well as how these genes and mechanisms are impacted by heat stress. This will enhance our understanding of heat stress response and adaptation, and facilitate future improvements of heat tolerance in layer chickens through genetic selection.

## 2. Materials and Methods

### 2.1. Experimental Design and Tissue Collection

Forty-eight W-36 female parent line laying hens (Hy-Line International, West Des Moines, IA, USA) were obtained at 18 weeks of age (pre-production) and acclimated for 6 weeks at 23 °C in Virginia Tech (VT). They were divided equally into control and heat-treated groups at 24 weeks of age (start of peak egg production). Birds were housed in individual battery cages with ad libitum access to mash layer feed and water in climate- and light-controlled rooms at VT. Heat-treated groups were exposed to cyclic heat for 4 weeks (from 24 weeks to 28 weeks of age), with daily heat cycles consisting of 7 h of elevated temperature at 35 °C and 17 h of ambient temperature at 30 °C. Control birds remained at 23 °C throughout the entire experiment. Birds were euthanized and the tissues were collected at 3 time points in 2-week intervals that evenly divided the 4-week experiment: acute heat stress (exposure to heat for 3 h), and chronic heat stress at 2 and 4 weeks of cyclic heat because previous studies have demonstrated that the transcriptomic response is quite different upon first and later exposures to heat [11,24,25]. At each time point, 1 cm cubes were dissected from the pectoralis major muscle and liver for 8 birds from the heat treatment group and 8 birds from the control group, diced and immediately placed in RNAlater (Sigma-Aldrich, St. Louis, MO, USA), transferred to Iowa State University, and stored at −80 °C until RNA isolation.

### 2.2. Total RNA Isolation

Total RNA was extracted from the pectoralis major muscle samples using a combination of phenol extraction from the mirVana miRNA Isolation Kit (Invitrogen, Carlsbad, CA, USA) followed by column purification from the RNAqueous Total RNA Isolation Kit (Invitrogen), using manufacturer’s instructions for each part of the isolation. Total RNA for the liver samples was extracted with the mirVana miRNA Isolation Kit using manufacturer’s instructions. Ambion DNA-free DNA Removal kit (Invitrogen) was used to remove contaminating DNA from RNA preparations. RNA concentrations were measured using a NanoDrop ND-1000 spectrophotometer (ThermoScientific, Waltham, MA, USA), and RNA integrity was assessed using the Agilent RNA Nano 6000 Kit on the Bioanalyzer 2100 (Agilent Technologies, Santa Clara, CA, USA). All samples used for cDNA library preparation had an RNA Integrity Number (RIN) score greater than 9.0 for the pectoralis major muscle and greater than 7.0 for the liver. 

### 2.3. RNA-Seq Design and cDNA Library Construction

For the RNA-Seq experiment, two factors were considered: treatment (heat-treated, control) and time (3 h, 2 weeks, 4 weeks), which formed six treatment groups (control 3 h, heat-treated 3 h, control 2 weeks, heat-treated 2 weeks, control 4 weeks, heat-treated 4 weeks). Each group had 4 randomly chosen biological replicates from the 8 harvested for each treatment by time group, for a total of 24 pectoralis muscle and 24 liver samples for RNA-Seq. The remaining samples (*n* = 24) were not used further in this study. When available, liver and muscle samples from the same birds were used (*n* = 14, 58%). A cDNA library for each sample was constructed using the Illumina TruSeq Stranded mRNA Library Prep Kit (Illumina Inc., San Diego, CA, USA) following the manufacturer’s standard protocol. Libraries were sequenced on the HiSeq 3000 platform (Illumina) to produce 100 bp paired-end reads at the Iowa State University’s DNA Facility (Iowa State University, Ames, IA, USA). Treatment groups were balanced across 2 lanes on the sequencing platform with a total of 12 libraries per lane for the pectoralis major muscle samples, and across 4 lanes with a total of 12 libraries per lane (6 libraries per lane were another tissue not included in this study) for the liver samples.

### 2.4. Sequence Read Quality Control, Mapping, and Counting

Raw read quality was assessed using the FastQC suite version 0.11.5 (http://www.bioinformatics.babraham.ac.uk/projects/fastqc/ (accessed on 31 December 2020)) for an initial quality check. Raw reads were filtered and trimmed with Trimmomatic (version 0.36) to remove reads containing adapter sequences and low quality reads [26]. The remaining high-quality reads were used as input for STAR (version 2.5.3a) alignment to the Galgal6a reference genome (Ensembl release 99) [27,28]. Using SAMtools, BAM files from the alignment were sorted by name, converted to SAM format, and used as input for HTSeq-count [29,30]. HTSeq counted the reads uniquely aligned to each gene in the Galgal6a Ensembl GTF annotation file. All bioinformatics software were executed with the default parameters unless otherwise stated.

### 2.5. Differential Expression Analysis

Relative similarity between transcriptomes was visualized with principal component analysis (PCA) plots generated by pcaExplorer (version 2.16.0) in R [31]. DEGs between treatments and times were obtained through analysis using edgeR (version 3.32.0) with the generalized linear model option [32]. Lowly expressed genes were first removed based on the criteria of less than 4 samples with more than 1 count per million. To minimize the effects of technical bias and sequencing depth variation, trimmed mean of M-values (TMM) method was utilized to normalize numbers of reads in edgeR [33]. Heat treatment and time were fitted as the main effects in the model. Thresholds for log2 fold change (log_2_FC) and false discovery rate (FDR, determined by Benjamini–Hochberg method [34]) were used to filter significant DEGs; genes with |log_2_FC| > 1 and FDR < 0.1 were defined as significant DEGs in each contrast.

### 2.6. Additional Analyses on DEGs

Gene Ontology (GO) terms from the DEGs were summarized using PANTHER [35]. With the lists of DEGs from the different contrasts, overlapping DEGs were identified using Venny (version 2.1, http://bioinfogp.cnb.csic.es/tools/venny/ (accessed on 31 December 2020)). Ingenuity Pathway Analysis (IPA, QIAGEN Inc., CA, USA) software combines information from their internal privately curated database with uploaded gene lists and corresponding log_2_FC to perform prediction of affected pathways, upstream and downstream effects. For this study, we used the “Diseases & Functions” feature to predict downstream effects from our DEGs lists [36]. Additional analyses were attempted with DEGs using DAVID [37] and Kyoto Encyclopedia of Genes and Genomes (KEGG) Mapper [38], but did not produce further results beyond that obtained with IPA due to the low number of annotated DEGs.

### 2.7. Correlation of RNA-Seq and qPCR Results

To validate the differential expression analysis, the Biomark HD system (Fluidigm Corporation, South San Francisco, CA, USA) was used to perform semi-high throughput microfluidic qPCRs on 192 × 24 Dynamic Array Integrated Fluidic Circuits (Fluidigm Corporation) using the same 24 RNA isolates used for RNA-seq. Genes for validation were chosen from the RNA-Seq DEGs lists to collectively represent all contrasts and varying expression levels. All primer pairs were designed in-house, produced by IDT (Integrated DNA Technologies, Coralville, IA, USA), and tested with traditional RT-qPCR for ability to yield a single product of a predicted size before running on the microfluidic qPCR system. To ensure high accuracy and specificity, only qPCR outputs with a single peak in the melting curve and sigmoidal amplification curves were used for validation. Additionally, the product produced from the RT-qPCR was sequenced to ensure that the intended target amplicon was produced. The list of 42 microfluidic qPCR primers used for RNA-Seq validation can be found in Supplementary Table S1. Average delta Ct values were calculated from triplicates using *HPRT1* as the reference gene for the muscle and *RPL4* as the reference gene for the liver for normalization. Microfluidic qPCR log_2_FC was calculated based on the delta-delta Ct method [39]. Pearson correlations between the log_2_FC from RNA-Seq and microfluidic qPCR were calculated in Microsoft Excel 2013 (Microsoft Corporation, Redmond, WA, USA). 

## 3. Results

### 3.1. Transcriptome Alignment, Mapping, and PCA Analysis

For the 24 muscle samples, 803 million 100-base paired-end reads were generated in total from 2 lanes in a single chip sequenced on the Illumina HiSeq 3000. The 24 liver samples generated 784 million 100-base paired-end reads from 2 lanes. After performing read quality control, there were on average 33 million paired-end reads per sample in the datasets for muscle and liver. The muscle sequence reads had an approximately 88% mapping rate by STAR alignment to the Galgal6 reference genome, which translated to approximate 61% coverage of the 24,356 genes listed in the Ensembl annotation. The liver sequence reads had higher mapping rate (94%) and gene coverage (64%) compared to the muscle samples. Detailed information for read alignment and mapping for each sample can be found in Supplementary Table S2. Prior to performing the differential gene expression analysis, PCA plots were generated for each of the seven contrasts for both tissues to ensure the contrasted factors can be separated by principal components (Supplemental Figure S1).

### 3.2. Effect of Heat Stress on Gene Expression in Egg-Producing Layers

**Figure 1:**
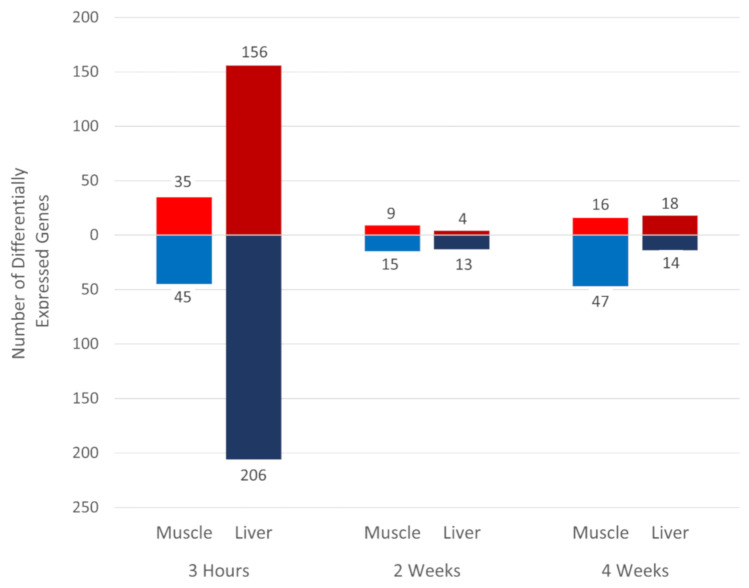
Number of differentially expressed genes (DEGs) for heat-control contrasts. Genes with |log_2_FC| > 1 and false discovery rate (FDR) < 0.1 were defined as significant DEGs in each contrast. Red = upregulated DEG counts and blue = downregulated DEG counts.

The gene expression estimated from normalized read counts for samples collected from the heat-treated group were compared to the time-matched control group at 3 h, 2 weeks, and 4 weeks after starting the cyclic heat treatment (Figure 1). DEGs were determined based on the significance threshold of having absolute log_2_FC > 1 and FDR < 0.1. The largest number of DEGs were observed at the acute heat stress time point of 3 h in both the muscle (*n* = 80) and liver (*n* = 362) tissue. In contrast, there were fewer liver DEGs for the chronic heat stress time points of 2 weeks (*n* = 17) and 4 weeks (*n* = 32). The pectoralis major muscle showed a large reduction in number of DEGs at the chronic heat-stress time points of 2 weeks (*n* = 24) and 4 weeks (*n* = 63). The list of DEGs for all three time-matched heat-control contrasts can be found in Supplemental Table S3 for muscle and Supplemental Table S4 for liver.

Using the heat-control contrast at 3 h, which had the largest number of DEGs for both tissues, GO terms for the acute heat stress DEGs were summarized. The top-level biological process GO terms showed similar order of enrichment for both liver and pectoralis major muscle (Supplemental Figure S2a). The GO term “cellular process” had the greatest number of DEGs, and this GO term was explored further. The GO term “cellular metabolic process” was the most common GO term within cellular processes (Supplemental Figure S2b).

**Figure 2:**
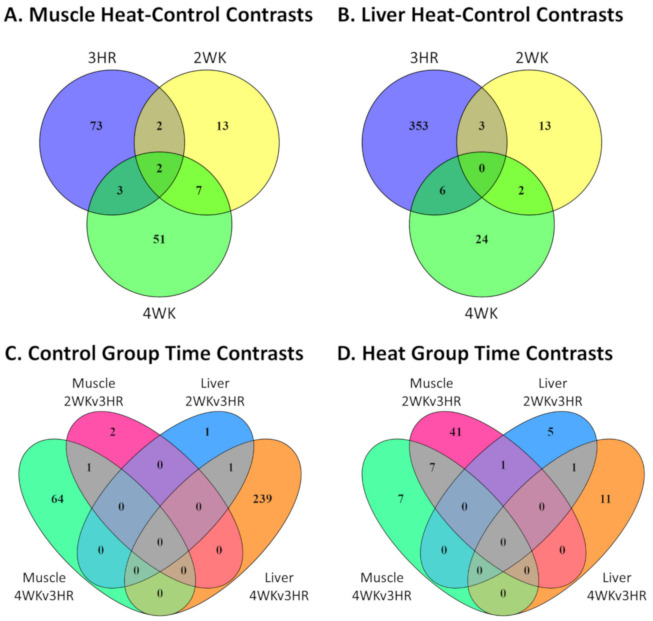
Overlap in differentially expressed genes (DEGs) for (**A**) heat-control contrasts for pectoralis major muscle tissue, (**B**) heat-control contrasts for liver tissue, (**C**) control group time contrasts for both tissues, and (**D**) heat-treated group time contrasts for both tissues. 3 HR = 3 h, 2 WK = 2 weeks, and 4 WK = 4 weeks.

Venn diagrams were constructed to find overlapping genes between the three heat-control contrasts for both pectoralis major muscle (Figure 2A) and liver tissues (Figure 2B). A total of 23 genes (12 from muscle and 11 from liver) were differentially expressed in at least 2 of the contrasts, and the annotation of these genes is provided in Table 1. Hydroxysteroid 17-beta dehydrogenase 7 (*HSD17B7*) and StAR-related lipid transfer domain containing 4 (*STARD4*) were found in all three heat-control contrasts for the pectoralis muscle. There was also one HSP from heat shock protein family A (hsp70) member 5 (*HSPA5*) that was differentially expressed in the liver at both 3 h and 4 weeks after starting heat treatment.

### 3.3. Gene Expression Changes in Layers during Early Production

**Figure 3:**
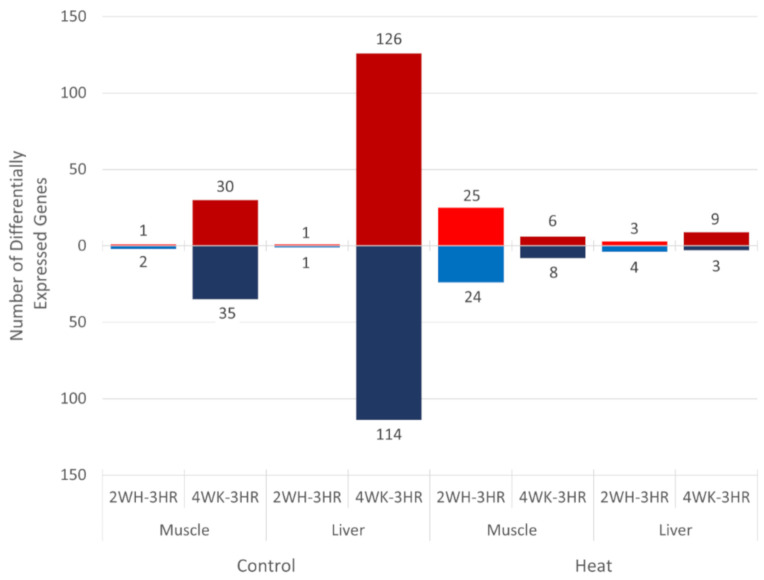
Number of differentially expressed genes (DEGs) for time contrasts for control and heat-treated groups. Genes with |log_2_FC| > 1 and FDR < 0.1 were defined as significant DEGs in each contrast. Red = upregulated DEG counts and blue = downregulated DEG counts. 3 HR = 3 h, 2 WK = 2 weeks, and 4 WK = 4 weeks.

To observe time-related changes in gene expression over the course of the 4-week experiment, 2- and 4-week gene expression were compared to 3 h gene expression within each treatment group (control and heat) and tissue (muscle and liver, Figure 3). The control group showed the greatest number of DEGs for both muscle (*n* = 65) and liver (*n* = 240) in the 4-week to 3 h contrast but very few DEGs in the 2-week to 3 h contrast. The heat treatment group had the greatest number of DEGs in the muscle tissue at 2 weeks compared to 3 h after starting heat treatment (*n* = 49) but few DEGs were observed in the other time contrasts within the heat-treated group. The list of DEGs for all time contrasts can be found in Supplemental Table S3 for muscle and Supplemental Table S4 for liver.

GO terms were summarized using the DEGs from the control group time contrast of 4-week compared to 3 h, which had the largest number of DEGs for both tissues. The top-level biological process GO terms showed similar order of enrichment for both liver and pectoralis major muscle (Supplemental Figure S2c). The GO term “cellular process” had the greatest number of DEGs, and the GO term was explored further. The GO term “cellular metabolic process” was the most common GO term within cellular processes (Supplemental Figure S2d).

The overlapping DEGs were identified for the time contrasts by constructing Venn diagrams: four muscle time contrasts (Figure 2C) and four liver time contrasts (Figure 2D). Fifteen overlapping DEGs were found, and only four DEGs were from the liver time contrasts (Table 2). A HSP from DnaJ heat shock protein family (Hsp40) member A4 (*DNAJA4*) was differentially expressed in the heat-treated pectoralis major muscle for both 2- and 4-week time contrasts, and pyruvate dehydrogenase kinase 4 (*PDK4*) was differentially expressed in both the pectoralis major muscle and the liver for the heat-treated 2-week to 3 h contrasts.

### 3.4. IPA Functional Predictions from Heat-Control and Control Group Time Contrasts and Corresponding Overlapping Genes 

**Figure 4:**
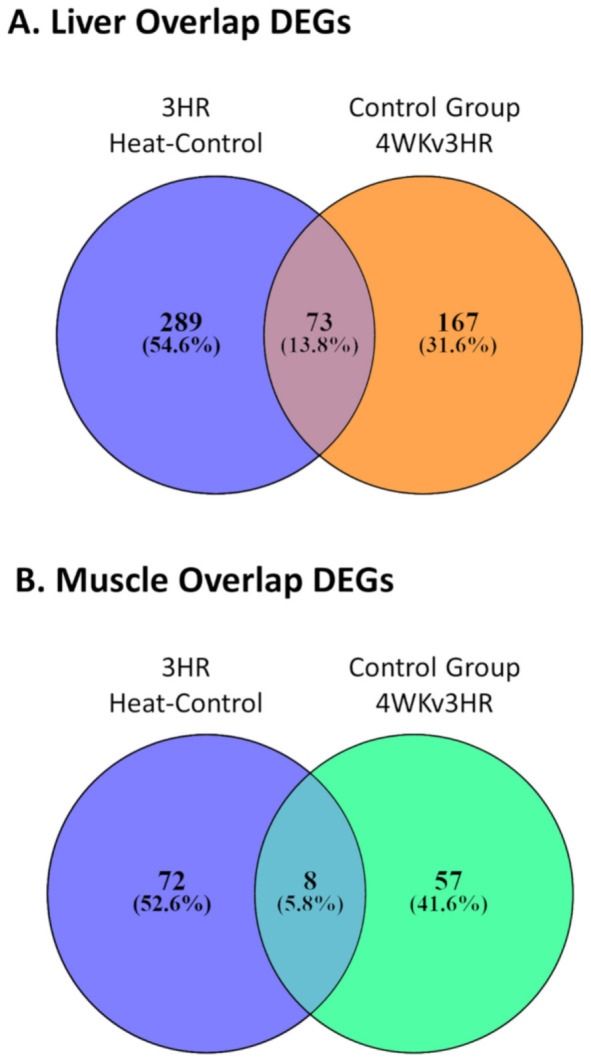
Results from analysis of the overlapping differentially expressed genes in the 3 h heat-control contrast and 4-week to 3 h control time contrast. (**A**) Overlap counts for liver and (**B**) overlap counts for pectoralis major muscle. 3 HR = 3 h, and 4 WK = 4 weeks.

**Figure 5:**
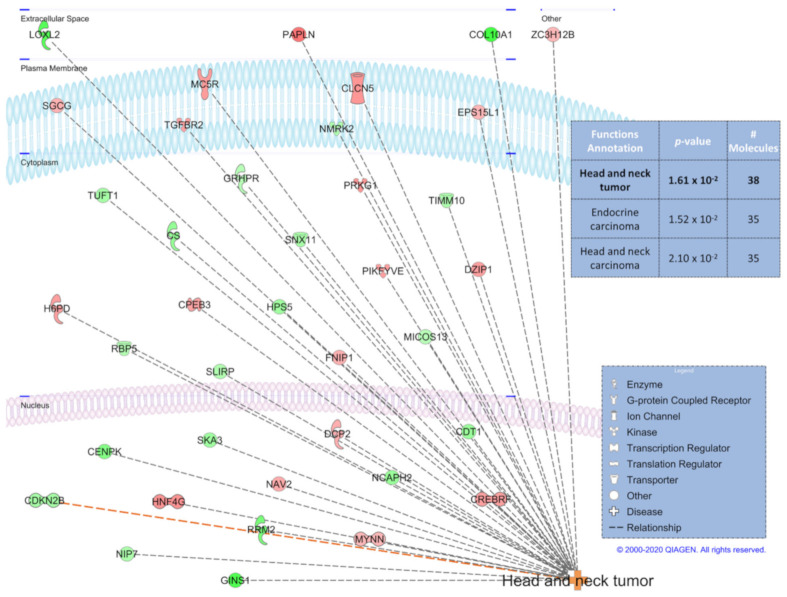
Ingenuity Pathway Analysis downstream function prediction of the overlapping differentially expressed genes from the liver 3 h heat-control contrast and 4-week to 3 h control time contrast. Table above the network legend shows the top 3 predictions and the bolded function prediction is displayed in the network.

From the summaries of GO term analysis for the contrasts that had the largest numbers of DEGs, we noticed similar enrichment of GO terms between the 3 h heat-control contrast and the control group 4-week to 3 h contrast. Overlapping the two contrasts for liver, we observed a sizable number (*n =* 73) of shared between the two contrasts (Figure 4A). Although not as numerous (*n =* 8), there were also overlapped DEGs in these same contrasts for the pectoralis major muscle (Figure 4B). For both tissues, all shared DEGs had the same direction of expression change for both contrasts (Supplementary Table S5). Downstream functional predictions were made for the liver DEGs using Ingenuity Pathway Analysis (IPA) software. From the liver 3 h heat-control contrast DEGs, IPA predicted a decrease in “hepatobiliary carcinoma” formation as the top prediction with 99 of the 362 DEGs (Supplementary Figure S3). From the control group 4-week to 3 h liver contrast, IPA predicted a decrease in “organismal death” with 42 of the 240 DEGs (Supplementary Figure S4). With 38 of the 73 overlapping liver DEGs from the two contrasts, IPA predicted these genes to work in concert to increase “head and neck tumor” formation (Figure 5). There were insufficient numbers of overlapping DEGs from the pectoralis major muscle contrasts to perform the same IPA downstream functional predictions as the liver. However, IPA was able to predict with 16 of the 80 DEGs from the pectoralis major muscle 3 h heat-control contrast DEGs that “synthesis of lipid” decreased (Supplementary Figure S5).

### 3.5. Correlation of RNA-Seq and qPCR Results

Forty genes (23 from muscle and 17 from liver) were selected from the RNA-Seq results and used for validation by qPCR. RNA-Seq estimated log_2_FC were compared against the qPCR estimated log_2_FC for the seven contrasts analyzed for each tissue. Correlation of gene expression between the two methods was estimated based on 280 pair-wise comparisons and a correlation value (R) of 0.76 was observed (Supplementary Figure S6).

## 4. Discussion

Although both pectoralis major muscle and liver play important roles in maintenance of metabolic homeostasis for laying hens, these two tissues are rarely analyzed in a single experiment. In this study, we took multiple approaches to analyze RNA-Seq data generated from 24 muscle tissues and 24 liver tissues collected during a 4-week heat stress experiment. Analysis of the dataset with PCA suggested that heat stress induced only moderate gene expression changes compared to the control group because the heat and control samples did not form tight clusters based on two principal components. Yet heat exposure using this 4-week protocol was shown to have a strong negative impact on egg production for heat-treated layers in a parallel heat stress experiment [7,8]. 

Our primary objective was to characterize changes in gene expression during acute and chronic heat stress of laying hens during early-stage egg production. As expected, muscle and liver tissues exhibited different responses to heat stress at the gene expression level. Under acute heat stress, the liver tissue mounted a large response altering the expression of more than 350 genes. Previous chicken liver transcriptome studies have also found gene expression changes at onset of heat stress [11,40]. However, we observed in this study that these changes in gene expression by the liver are short lived, as there were fewer DEGs observed for the chronic heat stress time points. In contrast to the liver, the muscle tissue had a milder response at the onset of heat stress, but changes in gene expression continued to occur throughout the chronic heat stress time points.

Not only are there differences in expression patterns for muscle and liver DEGs under heat stress, but the functions of these DEGs are also distinct despite both tissues having the GO term “cellular metabolic process” enriched. Muscle DEGs were more directly involved in energy metabolism, with *STARD4* and *HSD17B7* identified in all three heat-control contrasts. *STARD4* was also identified by IPA as a factor predicted to decrease “synthesis of lipid” in muscle based on the acute heat-control contrast DEGs. *STARD4* is best known for coding for protein with a role in maintaining cellular cholesterol homeostasis, and was previously identified as a DEG in chicken testes samples from an acute heat stress experiment [41,42]. *HSD17B7* had been shown in multiple chicken breeds to code for one of the factors controlling fat deposition and muscle growth rate [43,44,45]. Acyl-CoA synthetase bubblegum family member 2 (*ACSBG2*), stearoyl-CoA desaturase (*SCD*), and insulin induced gene 1 (*INSIG1*) were other DEGs identified in multiple heat-control contrasts within the muscle, and all three genes had been previously identified in various chicken tissues as having transcriptional changes in response to changes in energy metabolism, particularly the metabolism of fatty acids [44,46,47]. The muscle DEGs identified are in agreement with changes in glycogen and energy utilization that are known to occur in muscles in response to heat stress [18].

In the liver, more DEGs indirectly influence the metabolism by changing the expression of upstream regulators. Of the liver DEGs that were found in multiple heat-control contrasts, *HSPA5* and protein disulfide isomerase family A member 2 (*PDIA2*), which both codes for proteins involved with regulation of protein folding, have been identified as differentially expressed in other heat stress experiments [48,49]. Basic helix-loop-helix family member A15 (*BHLHA15*), which has a regulatory role in transcription, had also been identified as a liver DEG in a chicken heat stress experiment [25]. Lastly, ovochymase 2 (*OVCH2*), of which the cellular function is not well characterized beyond having endopeptidase activity, was identified as one of the genes responsible for impaired lipid utilization in liver tissue of riboflavin-deficient chicken embryos [50]. Even though glycogen is also stored in the chicken liver [51], our data did not suggest that direct changes in glycogen metabolism were occurring in liver in response to heat treatment. Instead, our study suggests that systemic changes were occurring that may have indirectly changed energy metabolism in the liver tissue.

Given that these layers started egg production shortly before initiation of the heat treatment, we postulate that the many DEGs in the time contrasts for the control group are representative of the natural gene expression changes that layers undergo during their rapid physiological adjustment towards peak egg production. These gene expression changes occurred after the 2-week time point, as nearly all the DEGs in the controls were identified from the 4-week to 3 h time contrast for both tissues. The summary of GO terms from the control group 4-week to 3 h time contrast DEGs showed the GO term “cellular metabolic process” as the most enriched GO term for both tissues. This suggests that part of the physiological adjustments included changes to the metabolic activity within the liver and pectoralis major muscle.

For the muscle tissue, two gene families, fibrinogen and vitellogenin, had multiple members with large fold changes. Both fibrinogen alpha (*FGA*) and gamma (*FGG*) chains were downregulated in the controls at the 4-week time point compared to the 3 h time point. Fibrinogen is best known in poultry for being involved with the formation of blood clots [52]. The downregulation of fibrinogen expression has been previously reported for muscle tissues collected from broilers with a muscular disorder [53]. The vitellogenin family is mostly known for its role in the ovary as the major precursor for yolk protein [54]. Both vitellogenin I (*VTG1*) and vitellogenin II (*VTG2*) were strongly downregulated in the 4-week to 3 h contrast in the control muscle tissue. However, a prior study of layers that had undergone ovariectomy found *VTG1* and *VTG2* downregulated in the liver tissue [55]. The downregulation of both fibrinogen and vitellogenin in the muscle tissue suggests conservation of local energy metabolism and reduction in protein synthesis in response to increased egg production in reproductive tissues.

For the liver tissue, calcium sensing receptor (*CASR*), lysyl oxidase like 2 (*LOXL2*), Hes family basic helix-loop-helix transcription factor 6 (*HES6*), GINS complex subunit 1 (*GINS1*), and ribonucleotide reductase regulatory subunit M2 (*RRM2*) were among the DEGs with large fold changes from the 4-week to 3 h time contrast of the control group. Unlike in mammals, *CASR* has the important role of maintaining calcium homeostasis in chickens in addition to regulating parathyroid hormone secretion [56]. Although *LOXL2* has been identified as a gene that codes for a key protein in the chicken eggshell membrane [57], *LOXL2* in liver probably has the role of biogenesis of connective tissues [58]. The roles of *HES6*, *GINS1*, and *RRM2* are not well studied in chickens, however, all three genes codes for proteins that are known to have important cellular functions in other organisms [59,60,61]. Both muscle and liver tissues had transcriptomic responses to the physiological changes of peak egg production, but just like the DEGs identified from the heat-control contrasts, control group time contrast DEGs in muscle likely had a direct impact on metabolic activity while the liver DEGs had an indirect impact.

The heat-treated group did not show the same time-related change in gene expression pattern as the control group. There were many more differentially expressed genes shared among the time-contrasts in the heat-treated tissues. Identified in multiple previous chicken heat stress experiments [25,41,48,62], *DNAJA4*, a HSP coding gene, was identified as a DEG in response to heat treatment for both the 2-week and 4-week compared to 3 h time contrasts in muscle. We found *PDK4*, a mitochondrial gene that codes for a metabolic protein, to be differentially expressed at the 2-week to 3 h time contrast for both muscle and liver, and *PDK4* has also been previously identified as a DEG in multiple chicken heat stress experiments [63,64]. Given that we had observed a strong transcriptomic response to the acute heat stress for both tissues in the 3 h heat-control contrast, gene expression had changed for the heat-treated group at the 3 h time point. However, the small number of DEGs observed for the 2-week and 4-week compared to 3 h time contrasts in the heat-treated group suggests that the developmental changes for layers reaching peak egg production observed for the control group had either been dampened or triggered prematurely by acute heat stress for the heat-treated group.

Based on novel comparisons of contrasts, we found that acute exposure to high ambient temperature had induced some of the same transcriptomic responses observed later in the control group as natural developmental changes coinciding with entering peak egg production. The comparison of 3 h acute heat-control contrast (*n =* 362 DEGs) against the control group 4-week versus 3 h time contrast (*n =* 240 DEGs) for liver showed an overlap of 73 DEGs. For muscle, the comparison of 3 h treatment-control contrast (*n =* 80 DEGs) and 4-week control group time contrast (*n =* 65 DEGs) showed an overlap of eight DEGs. Moreover, the log_2_FC had 100% agreement in direction of change in expression in the two contrasts for all overlapping DEGs from both tissues suggesting that the overlapping DEGs responded similarly to the two different stressors.

We propose that these overlapping DEGs represent a core transcriptomic response characterizing the organism’s attempt to maintain homeostasis when responding to stressors as diverse as heat and the major physiological demands of peak egg production. The IPA downstream functional prediction based on 38 of the 73 liver overlapping DEGs suggested an upregulation of tumor formation, which may seem like an irrelevant prediction. However, given the training data and analysis algorithm that IPA uses [36], this suggests that the functions of these overlapping DEGs have mostly been characterized in human cancer literature and not in poultry. We hypothesize that some of the key features associated with tumors, such as regulation of cell cycle and of metabolism [65], and stress response, are also the functions of the DEGs identified in the current study. Four of the genes previously discussed from the analysis of other contrast comparisons (*FGA*, *LOXL2*, *GINS1*, and *RRM2*) were also found in these overlapping DEGs, further supporting the idea that these overlapping DEGs may be part of a core group of genes, at least in layers, for maintenance of homeostasis during a variety of external stressors.

## 5. Conclusions

Through our analysis of transcriptome data generated from a 4-week heat stress experiment in layers, we have identified groups of genes that have not been previously described to function together in response to heat stress. Additionally, several genes were differentially expressed both in response to heat and in time-related changes in hens entering peak production. These genes represent a common core of genes that are candidates for contributing to metabolic resilience in chickens. Understanding gene function and applying this information in genetic selection programs may facilitate the development of chicken populations that are more robust to a variety of environmental and physiologic stressors, thus improving both production and welfare. 

## Figures and Tables

**Table 1:** Significantly differentially expressed genes found in heat-control contrasts (HvC) at multiple times. H = heat-treated group, C = control group, 3 HR = 3 h, 2 WK = 2 weeks, and 4 WK = 4 weeks. Red = upregulated and blue = downregulated.

Ensembl ID	Gene ID	Contrast: HvC	Tissue	Full Name and Known Function
ENSGALG00000000241	STARD4	3 HR, 2 WK, 4 WK	Muscle	StAR-Related Lipid Transfer Domain Containing 4, cholesterol homeostasis
ENSGALG00000001000	HSPA5	3 HR, 4 WK	Liver	Heat Shock Protein Family A (Hsp70) Member 5, protein folding
ENSGALG00000001749	ACSBG2	2 WK, 4 WK	Muscle	Acyl-CoA Synthetase Bubblegum Family Member 2, fatty acyl-CoA biosynthesis
ENSGALG00000005318	OVCH2	2 WK, 4 WK	Liver	Ovochymase 2, serine-type endopeptidase activity
ENSGALG00000005739	SCD	3 HR, 4 WK	Muscle	Stearoyl-CoA Desaturase, fatty acid biosynthesis
ENSGALG00000008349	LMCD1	2 WK, 4 WK	Muscle	LIM and Cysteine Rich Domains 1, transcription factor co-regulator
ENSGALG00000011376	ANKRD9	3 HR, 2 WK	Liver	Ankyrin Repeat Domain 9, integral membrane protein
ENSGALG00000012610	CTSV	3 HR, 4 WK	Muscle	Cathepsin V, collagen chain trimerization
ENSGALG00000017394	INSIG1	2 WK, 4 WK	Muscle	Insulin Induced Gene 1, cholesterol metabolism, lipogenesis, and glucose homeostasis
ENSGALG00000017804	Y_RNA	3 HR, 4 WK	Liver	Small non-coding RNA, component of the Ro60 ribonucleoprotein, target of autoimmune antibodies
ENSGALG00000029006	SYPL1	2 WK, 4 WK	Muscle	Synaptophysin Like 1, transporter activity for synaptic vesicle
ENSGALG00000031306	BHLHA15	3 HR, 4 WK	Liver	Basic Helix-Loop-Helix Family Member A15, controls transcriptional activity of myoblast differentiation
ENSGALG00000037156	Novel	2 WK, 4 WK	Muscle	Orthologue = zgc:136493, zinc-finger protein, predicted to be involved with oxidation-reduction process
ENSGALG00000037852	HSD17B7	3 HR, 2 WK, 4 WK	Muscle	Hydroxysteroid 17-Beta Dehydrogenase 7, biosynthesis of sex steroids
ENSGALG00000040070	PDIA2	3 HR, 2 WK	Liver	Protein Disulfide Isomerase Family A Member 2, protein folding
ENSGALG00000049504	Novel	2 WK, 4 WK	Liver	Orthologue = SLC2A4, Solute Carrier Family 2 Member 4, facilitated glucose transporter
ENSGALG00000050857	PTPN20	3 HR, 4 WK	Liver	Protein Tyrosine Phosphatase, Non-Receptor Type 20, protein tyrosine phosphatase
ENSGALG00000051251	Novel	2 WK, 4 WK	Muscle	Orthologue = H2B, human histone cluster 1, class H2B, histone
ENSGALG00000051816	Novel	3 HR, 4 WK	Muscle	LncRNA
ENSGALG00000052045	Novel	2 WK, 4 WK	Muscle	LncRNA
ENSGALG00000053112	Novel	3 HR, 4 WK	Liver	Orthologue = FRMPD2, FERM and PDZ Domain Containing 2, cell polarization
ENSGALG00000053926	Novel	3 HR, 4 WK	Liver	LncRNA
ENSGALG00000054926	Novel	3 HR, 2 WK	Liver	Unknown

**Table 2:** Significantly differentially expressed genes found in multiple within-treatment time contrasts. H = heat-treated group, C = control group, 3 HR = 3 h, 2 WK = 2 weeks, and 4 WK = 4 weeks. Red = upregulated and blue = downregulated.

Ensembl ID	Gene ID	Contrast	Tissue	Full Name and Known Function
ENSGALG00000001749	ACSBG2	C_2 WKv3 HR, C_4 WKv3 HR	Muscle	Acyl-CoA Synthetase Bubblegum Family Member 2, fatty acyl-CoA biosynthesis
ENSGALG00000006009	Novel	H_2 WKv3 HR, H_4 WKv3 HR	Muscle	Orthologue = PODXL2, Podocalyxin Like 2, CD34 family of cell surface transmembrane protein
ENSGALG00000008912	ABCB1	H_2 WKv3 HR, H_4 WKv3 HR	Liver	ATP Binding Cassette Subfamily B Member 1, multidrug resistance
ENSGALG00000009700	PDK4	H_2 WKv3 HR	Muscle, Liver	Pyruvate Dehydrogenase Kinase 4, mitochondrial protein in regulation of glucose and fatty acid metabolism
ENSGALG00000014554	ATN1	H_2 WKv3 HR, H_4 WKv3 HR	Muscle	Atrophin 1, transcriptional corepressor, promotes vascular smooth cell migration
ENSGALG00000023819	Novel	H_2 WKv3 HR, H_4 WKv3 HR	Muscle	Orthologue = HSPB11, Heat Shock Protein Family B (Small) Member 11, SHH signaling
ENSGALG00000036850	DNAJA4	H_2 WKv3 HR, H_4 WKv3 HR	Muscle	DnaJ Heat Shock Protein Family (Hsp40) Member A4, unfolded protein binding
ENSGALG00000036956	Novel	H_2 WKv3 HR, H_4 WKv3 HR	Muscle	Mitochondrial rRNA
ENSGALG00000041459	DDIT4	H_2 WKv3 HR, H_4 WKv3 HR	Muscle	DNA Damage Inducible Transcript 4, response to cellular energy levels and cellular stress from hypoxia and DNA damage
ENSGALG00000049873	Novel	C_2 WKv3 HR, C_4 WKv3 HR	Liver	LncRNA
ENSGALG00000051251	Novel	H_2 WKv3 HR, H_4 WKv3 HR	Muscle	Orthologue = H2B, human histone cluster 1, class H2B, histone

## Data Availability

All sequencing data from this study have been deposited in ArrayExpress (https://www.ebi.ac.uk/arrayexpress/ (accessed on 31 December 2020)) with accession number E-MTAB-7479 for pectoralis muscle and E-MTAB-7480 for liver.

## Supplementary Materials

The following are available online at https://www.mdpi.com/2073-4425/12/2/255/s1, Figure S1: Principle Component Analysis Plots, Figure S2: Gene Ontology Terms Summary, Figure S3: Liver HvC 3 HR Ingenuity Pathway Analysis, Figure S4: Liver C 4 WKv3 HR Ingenuity Pathway Analysis, Figure S5: RNA-Seq Validation, Table S1: qPCR Primers, Table S2: RNA-Seq Data Information, Table S3: Muscle DEG Lists, Table S4: Liver DEG Lists, Table S5: Time Overlap DEGs.
