# Supplementary material for: Transcriptome Response of Liver and Muscle in Heat-Stressed Laying Hens
Source: Genes (Basel). 2021 Feb 10;12(2):255. doi: 10.3390/genes12020255 (PMC7916550; doi:10.3390/genes12020255)

1. Pectoralis major muscle tissue at 3 hours, heat-treated vs. control samples


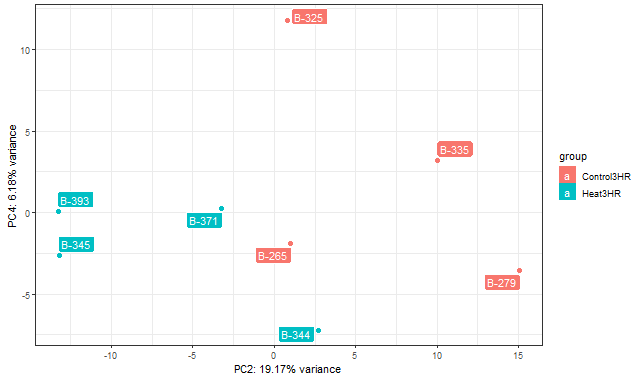


1. Pectoralis major muscle tissue at 2 weeks, heat-treated vs. control samples


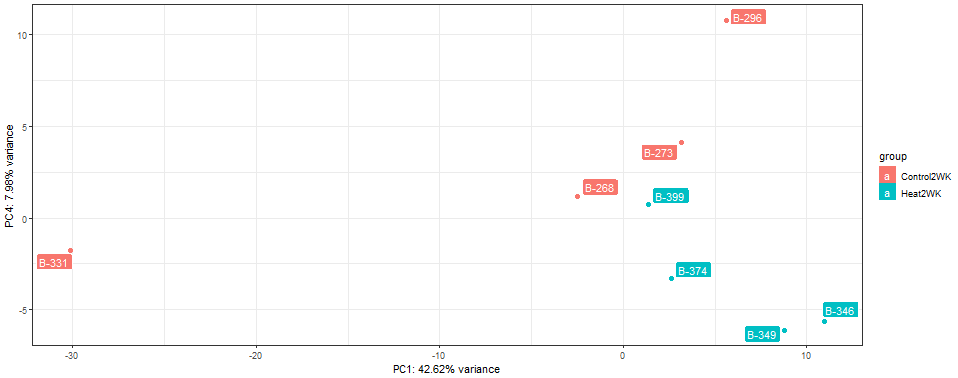


1. Pectoralis major muscle tissue at 4 weeks, heat-treated vs. control samples


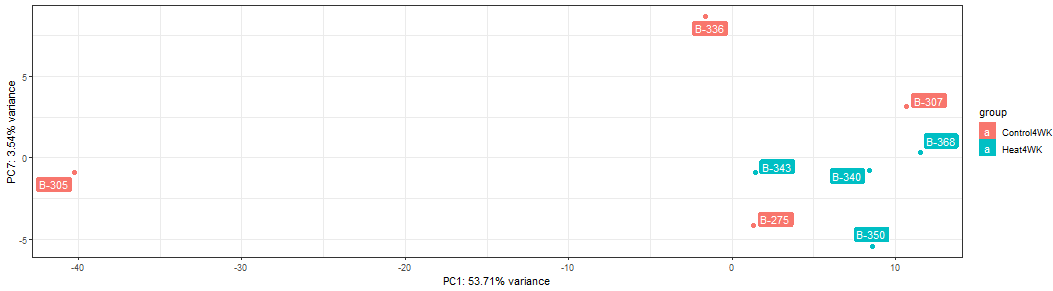


1. Pectoralis major muscle tissue control samples, 2 weeks vs. 3 hours


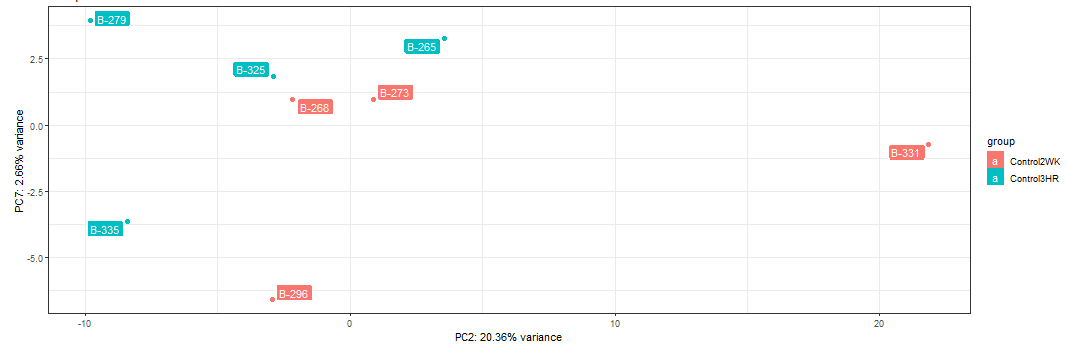


1. Pectoralis major muscle tissue control samples, 4 weeks vs. 3 hours


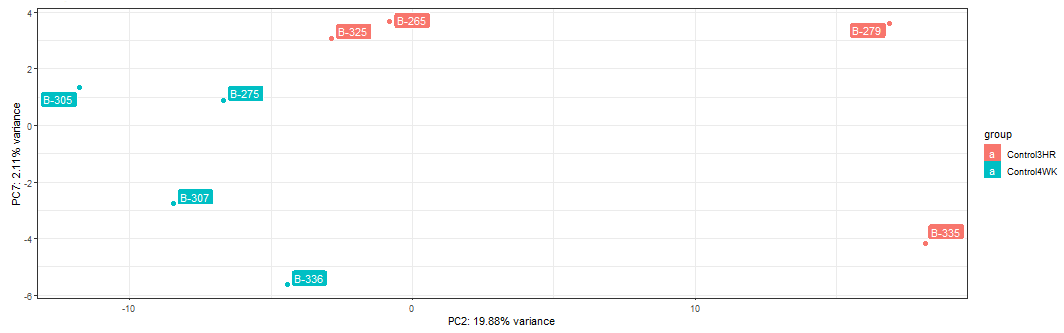


1. Pectoralis major muscle tissue heat-treated samples, 2 weeks vs. 3 hours


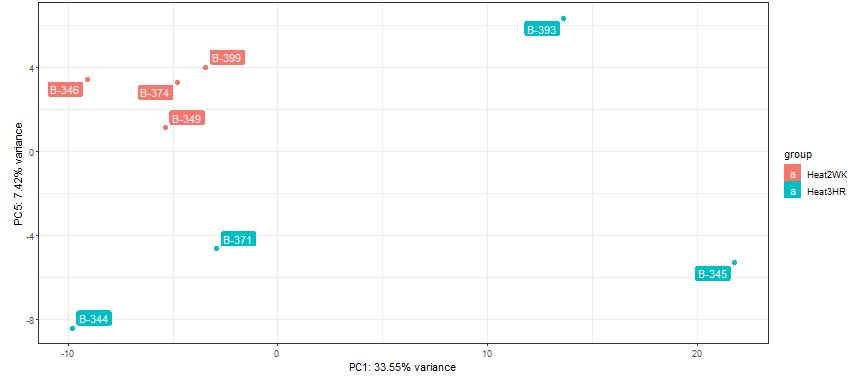


1. Pectoralis major muscle tissue heat-treated samples, 4 weeks vs. 3 hours


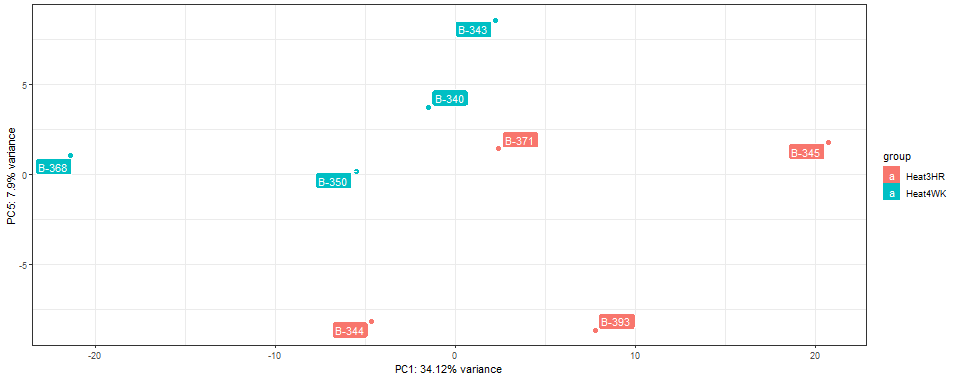


1. Liver tissue at 3 hours, heat-treated vs. control samples


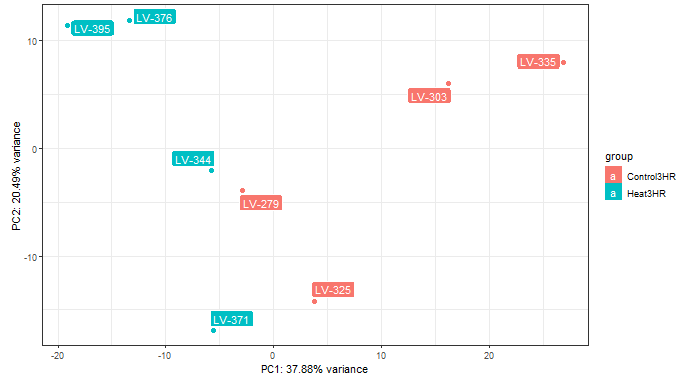


1. Liver tissue at 2 weeks, heat-treated vs. control samples


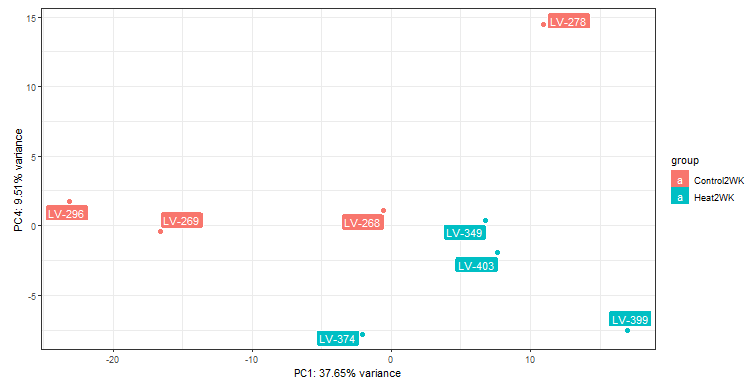


1. Liver tissue at 4 weeks, heat-treated vs. control samples


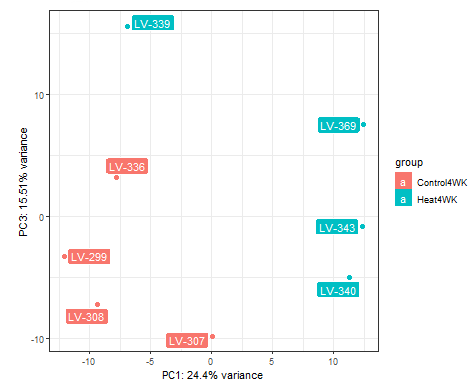


1. Liver tissue control samples, 2 weeks vs. 3 hours


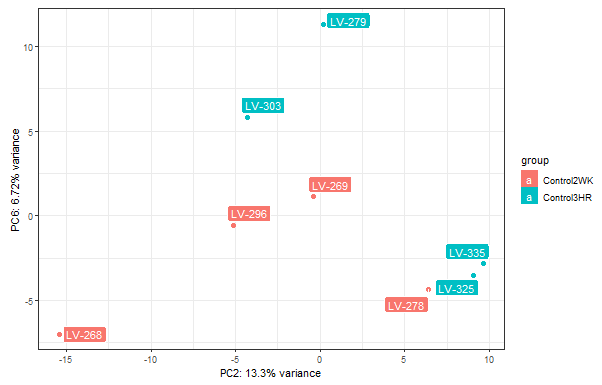


1. Liver tissue control samples, 4 weeks vs. 3 hours


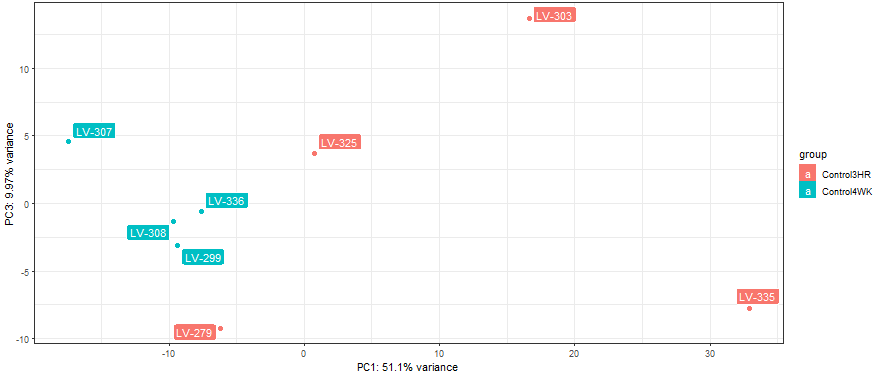


1. Liver tissue heat-treated samples, 2 weeks vs. 3 hours


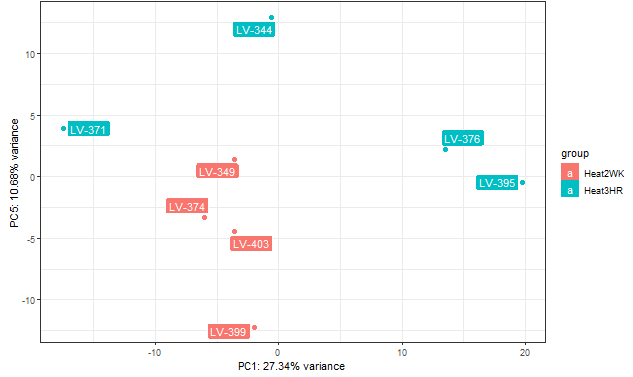


1. Liver tissue heat-treated samples, 4 weeks vs. 3 hours


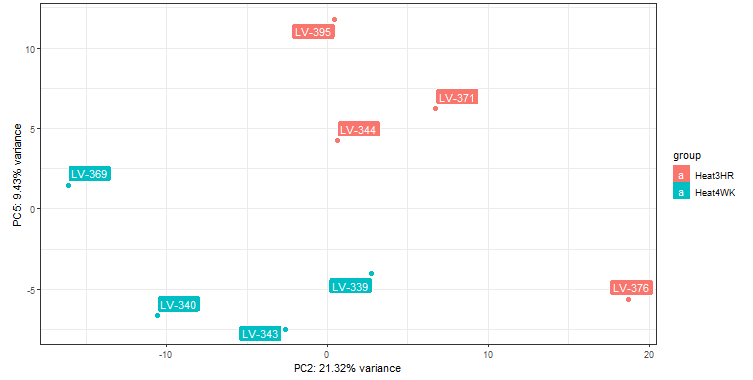

Supplement: Supplementary file 1 [file genes-12-00255-s001.zip › Supplemental Figures and Tables v6 - 20210128/Figure S1 - PCA Plots.docx]
