# Supplementary material for: Transcriptome Response of Liver and Muscle in Heat-Stressed Laying Hens
Source: Genes (Basel). 2021 Feb 10;12(2):255. doi: 10.3390/genes12020255 (PMC7916550; doi:10.3390/genes12020255)
Supplement: Supplementary file 1 [file genes-12-00255-s001.zip › Supplemental Figures and Tables v6 - 20210128/Figure S2 - GO Terms Summary.docx]

1. Heat-treated vs. control samples at 3 hours, PANTHER GO-Slim Biological Process
2. Heat-treated vs. control samples at 3 hours, PANTHER GO-Slim Biological Process, Level 1: Cellular Process
3. Control samples 4 weeks vs. 3 hours, PANTHER GO-Slim Biological Process
4. Control samples 4 weeks vs. 3 hours, PANTHER GO-Slim Biological Process, Level 1: Cellular Process
