# Supplementary figures and images for: Transcriptome Response of Liver and Muscle in Heat-Stressed Laying Hens
Source: Genes (Basel). 2021 Feb 10;12(2):255. doi: 10.3390/genes12020255 (PMC7916550; doi:10.3390/genes12020255)

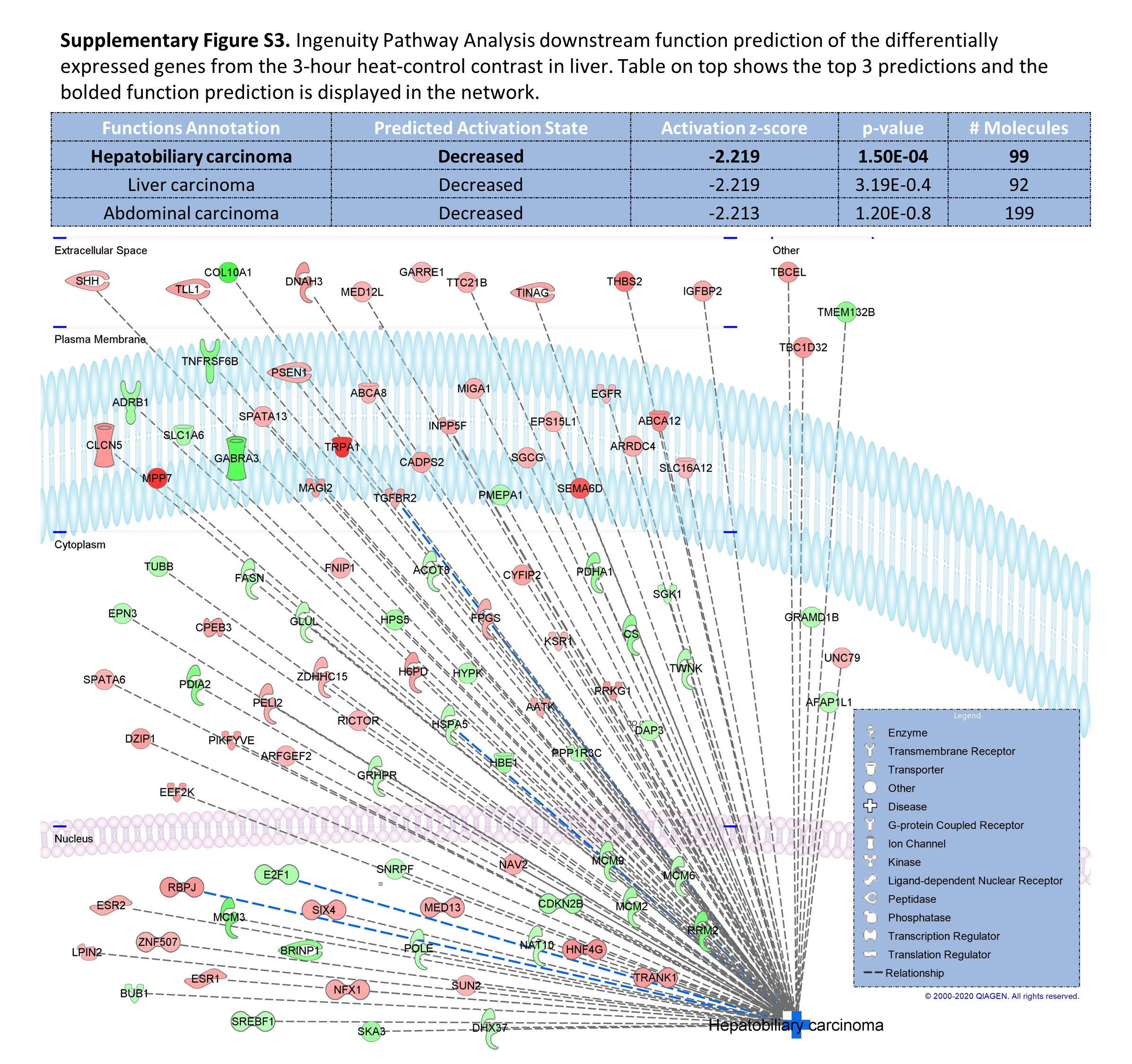

Supplement: Supplementary file 1 [file genes-12-00255-s001.zip › Supplemental Figures and Tables v6 - 20210128/Figure S3 - Liver HvC 3HR IPA.tif]

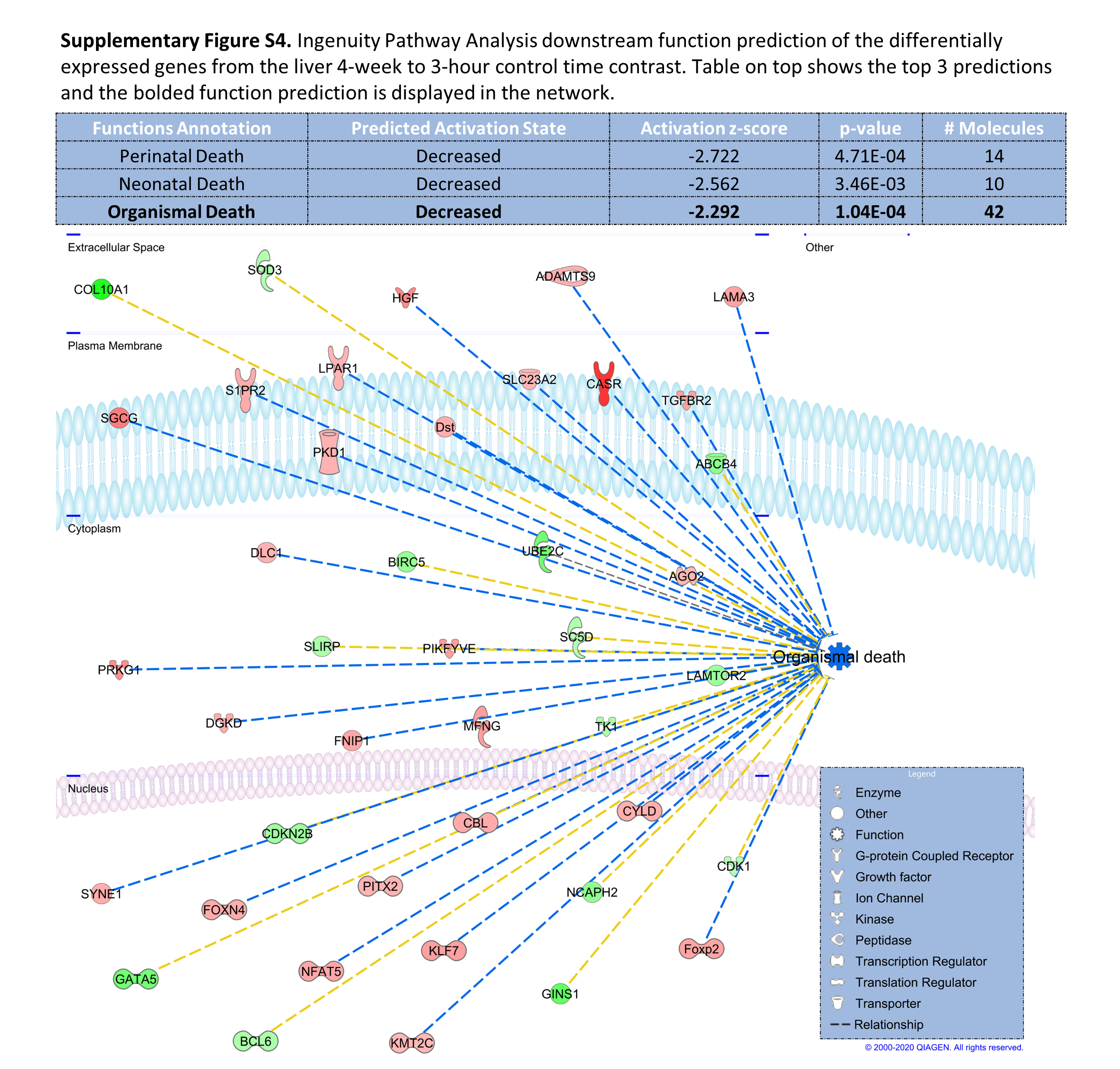

Supplement: Supplementary file 1 [file genes-12-00255-s001.zip › Supplemental Figures and Tables v6 - 20210128/Figure S4 - Liver C 4WKv3HR IPA.tif]

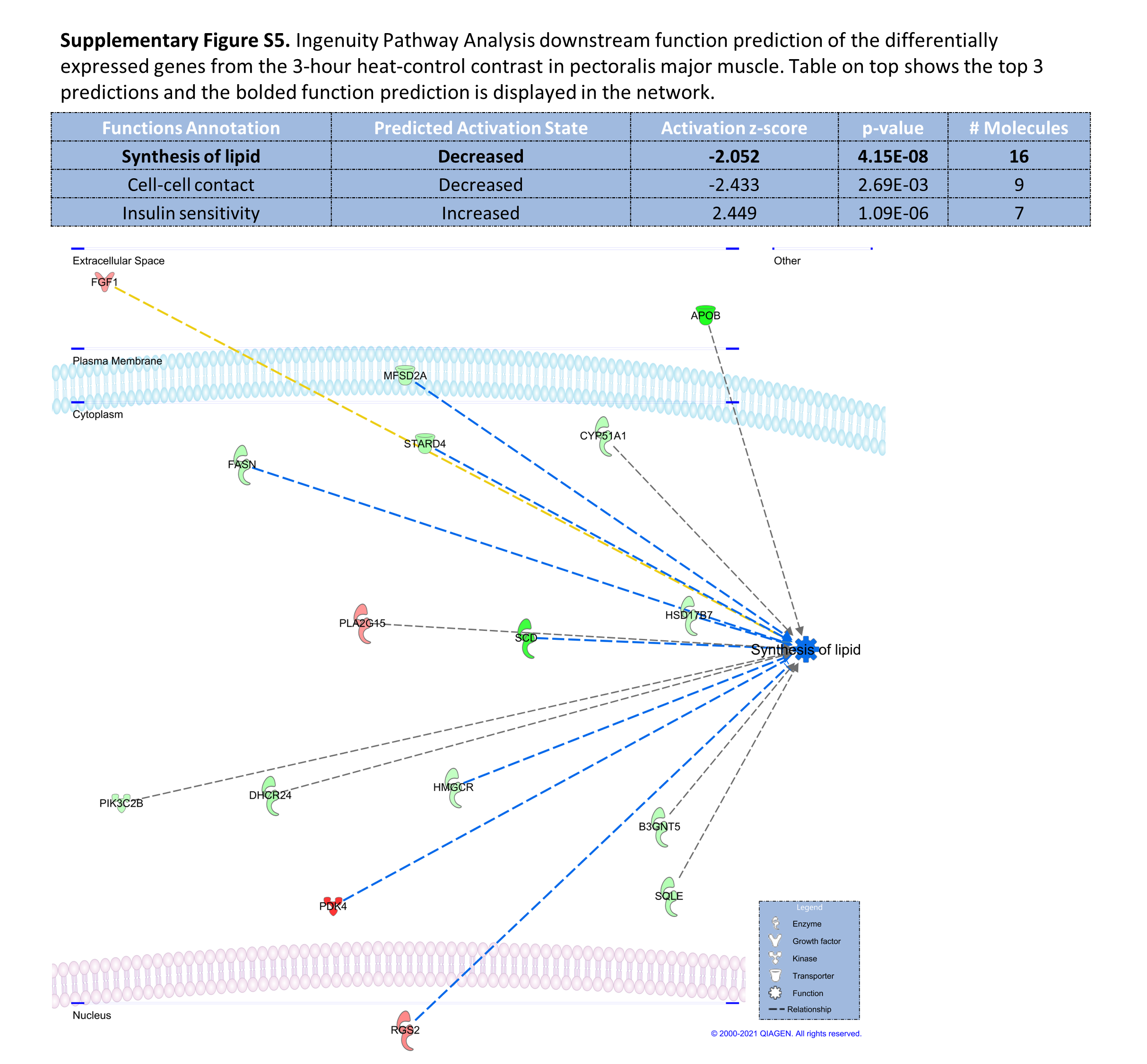

Supplement: Supplementary file 1 [file genes-12-00255-s001.zip › Supplemental Figures and Tables v6 - 20210128/Figure S5 - Muscle HvC 3HR IPA.tif]

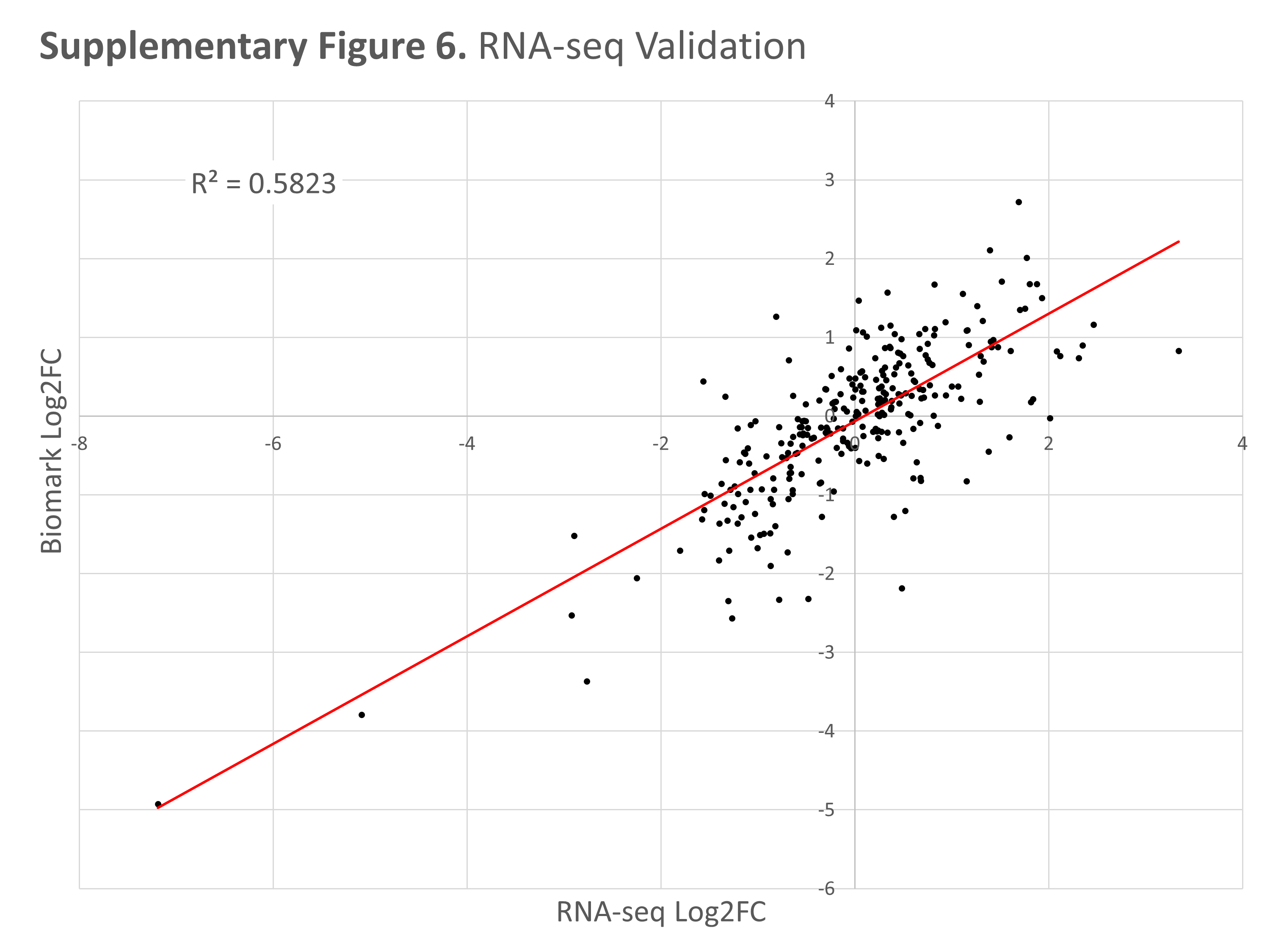

Supplement: Supplementary file 1 [file genes-12-00255-s001.zip › Supplemental Figures and Tables v6 - 20210128/Figure S6 - RNA-Seq Validation.tif]
